# Exploring intergenerational differences in burnout and how they relate to work engagement, norms, and values: a mixed-methods study

**DOI:** 10.3399/bjgpopen18X101637

**Published:** 2019-04-03

**Authors:** Carolien De Maeyer, Birgitte Schoenmakers

**Affiliations:** 1 Junior Doctor, Academic Centre of General Practice, Department of Public Health and Primary Care, University of Leuven, Leuven, Belgium; 2 Professor, Academic Centre of General Practice, Department of Public Health and Primary Care, University of Leuven, Leuven, Belgium

**Keywords:** burnout, generations, ethics, general practice, primary care

## Abstract

**Background:**

Burnout has been on the rise in recent years. Is this increasing prevalence due to changing working circumstances, or also to a changing societal context?

**Aim:**

The aim of the study was to explore intergenerational differences in burnout.

**Design & setting:**

The study used a mixed quantitative and qualitative design, and tested the theory of the job demands–resources model (JD-RM). The target group of this research was the working population in Belgium, aged between 21–65 years. The study was performed in a public setting with people recruited through social media.

**Method:**

A quantitative web survey was distributed among the participants to explore the prevalence of burnout, work ethic, work engagement, and norms and values. The second part of the study used a focus group technique to explore in depth the eight statements that were formulated from the quantitative survey.

**Results:**

A total of 309 people participated in the web survey and 21.5% met the criteria for 'burnout', while 22.6% scored high on the criteria for 'engagement'. It was found that 12.6% of all men and 3.0% of all women could not identify with the portrait of 'equivalence’. The value 'benevolence' was positively rated by 100%. In the youngest generation, 71.3 % identified with the portrait of ‘stimulation', while 70.8% of older men identified with the portrait ‘traditions’. The results also revealed that 43.1% of the younger generation agreed with the statement: 'I often think I would be more successful if I gave up certain pleasures'

**Conclusion:**

Burnout is more common among young people. In the older generation, a good ability to put things into perspective, a good balance between work and leisure, and a strong sense of tradition appear to be resources to aid against burnout. The younger generation often deals with stressors. This younger generation appears to have a higher work ethic and commitment.

## How this fits in

Intergenerational differences always led to conflicts; one ongoing conflict is the assumption of the older generation that the younger generation is more susceptible to burnout because they have a lower work ethic and too many other engagements. This study shows that the reality is more complicated. GPs should be aware of the intergenerational differences in the occurrence of burnout in order to provide more efficient support to their patients. 

## Introduction

The phenomenon of burnout has been on the rise in recent years.^1,2^ The feeling of being 'exhausted', 'empty', and ‘burned out' is something with which many people seem to be able to identify, to a greater or lesser extent.^2,3^ Clinically, burnout is a syndrome characterised by a triad of symptoms: emotional exhaustion; cynicism with regard to work; and reduced personal competence. The condition arises in case of prolonged exposure to emotional and interpersonal work demands, and when personal resources are vulnerable.^4,5^ Although initially described only in the case of contract jobs, in recent years people have become aware that the problem also occurs within other professional categories.^3,6^ The economic and personal consequences of burnout should not be underestimated, ranging from long-term absenteeism to psychiatric conditions.^1,7^ In order to better understand the concept of burnout, Demerouti *et al* formulated the JD-RM.^4,8^ This is a theoretical framework in which two types of job characteristics are defined, namely job demands and job resources. Job demands are the physical, psychological, social, or organisational aspects of the work that require sustained physical or mental effort, and are associated with certain physical and psychological costs. Job resources are the physical, psychological, social, or organisational characteristics of the work that ensure that you achieve your work goals, reduce job demands, and/or stimulate personal growth and development.^9,10^ In addition to this model, Schaufeli *et al* developed in 2004 an additional dimension, defined as 'engagement'.^11,12^

One question is whether the increasing burnout prevalence is owing to changing working circumstances and context, or whether it is also owing to a changing societal context for the younger generation. The aim of this study was to apply the theoretical framework of the JD-RM to the values, norms, and work ethic of the current working population. Therefore, two research questions were formulated:

1. Does the prevalence of burnout differ between generations?
2. Do intergenerational differences in work ethic and values and norms explain the differing burnout prevalence?

## Method

### Population

The target group of this research was the Flemish working population aged 21–65 years. No exclusion criteria were formulated. Participants were recruited through social media and public announcements.

### Design and outcome measures

The study had a mixed qualitative and quantitative, cross-sectional design without follow-up. The study consisted of two parts. In the first part, four quantitative questionnaires — measuring work ethic, values and norms, engagement, and burnout — were distributed among the participants.

Based on the results of part one, eight statements were formulated and presented in the second qualitative study section to three focus group: one focus group per generation, each consisting of eight participants.

The quantitative survey in the first part relied on four validated questionnaires. 'Burnout' was measured and determined using the Utrecht Burnout General Purpose Scale (UBOS-A). This version is a validated translation of the Maslach Burnout Inventory, and consists of 15 items addressing the three core symptoms of burnout. Each item of the UBOS-A is scored on a 7-point Likert scale, ranging from 0 (‘never’) to 6 (‘always/daily’).^13,14^ Cut-offs for burnout were calculated as the total sum score and the sum scores on the subscales exhaustion, emotional distance, competence: respectively, higher than 2, 19 (exhaustion); 1, 99 (emotional distance); 3, 76 (competence).

'Engagement' was defined as positive work-related state of mind characterised by vigour, dedication, and absorption. The Utrecht shortened Engagement Scale (UWES-9) was used to measure this outcome. This is a 9-item questionnaire with answer options along a 7-point Likert scale.^5,13^

'Work ethic' was assessed via the Protestant Work Ethic Scale, as per Mirels *et al*, translated into and validated in Dutch.^9^ Work ethic is defined as the belief that hard work and diligence have a moral benefit, and an ability to strengthen character and individual abilities. The scale consists of 19 theorems and is scored on a 6-point Likert scale, ranging from 1 (‘Do not agree at all’) to 6 ('Completely agree').^15^ There was no cut-off determined.

Finally, 'values' (general guidelines and standards) and 'norms' (culturally established rules) were measured via the Portrait Values Questionnaire (PVQ), in the version adapted for the European Social Survey (2014–2015). This Dutch questionnaire consists of 21 portraits that address the 10 basic human values, as defined by Schwartz *et al*. For each item, the responders indicate the extent to which the outlined portrait matches up to their own ‘portrait’. The results were scored on a 6-point Likert scale, ranging from 1 (‘Looks very much like me’) to 6 (‘Does not look like me at all’).^15–17^

All the questionnaires were drafted and implemented in the highly secured environment of the Limesurvey programme of the University of Leuven in Belgium. A paper version was available to the responders at the time of the survey.

After completion of the first quantitative study section, results were collected and analysed (univariate) via Microsoft Excel Professional Plus 2016. Based on the results of this first study section, the research group formulated, discussed, and adjusted (in a 'grounded theory' framework)^18^ statements. The statements departed from the presumption that personal values, norms, and work ethic affect the balancing of job demands and resources (the JD-RM).

The research group composed the statements addressing remarkable observations and representing the view of the participants on engagement, work ethic, and values and norms. Remarkable observations were defined as: significantly differing between groups (age and/or sex); or not in agreement with common reality or research. Significantly differing was determined as ≥60% of the participants marked ‘agreed’ or ‘disagreed’ as answer.

In this second study, section participants were randomly selected (equally distributed over all age groups) from the total study population and asked to participate in the focus groups. Inclusion criteria were being part of the working labour force, and having a permanent job. The exclusion criterium was a history of burnout or other moderate to severe psychological problems (such as depression or personality disorder). The participants were divided into three age categories: a group aged 21–35 years; a group aged 36–50 years; and a group aged 51–65 years. The groups each consisted of eight participants, with mutual anonymity.

This study section was performed following an adapted Delphi method: the consequent rounds of discussion, adaptation, and feedback on the statements were carried out in a digital (virtual) discussion group. The participants commented on the statements, and graded the importance and appropriateness to their generation of each statement. After each round, the responders' comments were collected, analysed (coded), and discussed in the research group according to the grounded theory method.^18^ The research group discussed the content of the comments, reformulated and recomposed them by consent and agreement, and then proposed the adapted version to the members of the focus group in a second round. After this second round, the research group again collected, analysed and adapted the comments of the participants. A third round was provided in case of major incongruities, disagreements, or misconceptions arising in the discussion groups.

## Results

### Web survey: quantitative section

The results of the web survey can be seen in Tables 1 and 2.

**Table 1.** The relation between burnout, work engagement, and norms and values: are there intergenerational differences?

	Burnout		No burnout		Total
**Sex**	**%**	***n***	**%**	***n***	***n***
Male	19.6	11	80.4	45	56
Female	29.3	17	70.7	41	58
Total	24.6	28	75.4	86	114
Male	22.6	7	77.4	24	31
Female	11.8	2	88.2	15	17
Total	18.8	9	81.3	39	48
Male	4.5	1	95.5	21	22
Female	36.4	4	63.6	7	11
Total	15.2	5	84.8	28	33
Total	21.5	42	78.5	153	195

**Table 2.** Score on engagement by age group and sex

Age group	Sex	Very low, %	*n*	Low, %	*n*	Average, %	*n*	High, %	*n*	Very high, %	*n*	Total, *n*
25–35 years	Male	7.1	4	25.0	14	41.1	23	25.0	14	1.8	1	56
	Female	3.4	2	17.2	10	50.0	29	29.3	17	0.0	0	58
	Total	5.3	6	21.1	24	45.6	52	27.2	31	0.9	1	114
36–50 years	Male	3.2	1	16.1	5	64.5	20	16.1	5	0.0	0	31
	Female	11.8	2	5.9	1	70.6	12	11.8	2	0.0	0	17
	Total	6.3	3	12.5	6	66.7	32	14.6	7	0.0	0	48
51–65 years	Male	4.5	1	4.5	1	72.7	16	18.2	4	0.0	0	22
	Female	0.0	0	45.5	5	45.5	5	0.0	0	9.1	1	11
	Total	3.0	1	18.2	6	63.6	21	12.1	4	3.0	1	33
End total		5.1	10	18.5	36	53.8	105	21.5	4	1.0	2	195

A total of 309 people participated in the web survey and 222 of them completed the four questionnaires. Missing data were reported in 27 cases. A total of 61.7% of the responders were aged 21–35 years, 21.6% between 36–50 years, and 16.7% between 51–65 years. The average age was 35 years. A total of 87.8% of the responders had a permanent job (Table 1).

A total of 21.5% of the responders met the criteria for 'burnout': 24.6% of the youngest generation; 18.8% of those aged 36–50 years; and 15.2% of the oldest generation. A total of 17.43% of all male participants and 26.74% of all female participants met the criteria for 'burnout', while 22.6% of the responders scored high to very high on the criteria for 'engagement' (28.1% of the youngest generation, 14.6% of those aged 36–50 years and 15.2% of the oldest generation), as shown in Table 2.

'Values' and 'norms' were investigated with reference to the portraits drawn in PVQ (Table 3).

**Table 3.** Degree of identification with portraits of equivalence, benevolence, stimulation, and traditions, respectively, by age group

Equivalence	Degree of identification with portrait^a^					
Age groups	1	2	3	4	5	6
25–35 years, %	15.6	45.3	25.0	6.3	7.8	0.0
36–50 years, %	16.1	48.4	19.4	9.7	3.2	3.2
51–65 years, %	37.5	54.2	4.2	4.2	0.0	0.0
Total, %	20.2	47.9	19.3	6.7	5.0	0.8

Benevolence	Degree of identification with portrait^a^			
Age groups	1	2	3	5
25–35 years, %	48.4	43.8	7.8	0.0
36–50 years, %	35.5	38.7	19.4	6.5
51– 65 years, %	41.7	50.0	8.3	0.0
Total, %	43.7	43.7	10.9	1.7

Stimulation	Degree of identification with portrait^a^					
Age groups	1	2	3	4	5	6
25–35 years, %	6.3	32.8	31.3	21.9	7.8	0.0
36–50 years, %	16.1	9.7	41.9	19.4	12.9	0.0
51–65 years, %	4.2	29.2	25.0	29.2	8.3	4.2
Total, %	8.4	26.1	32.8	22.7	9.2	0.8

Traditions	Degree of identification with portrait^a^					
Age groups	1	2	3	4	5	6
25–35 years, %	3.1	23.4	31.3	15.6	17.2	9.4
36–50 years, %	6.5	29.0	25.8	3.2	29.0	6.5
51–65 years, %	0.0	54.2	16.7	12.5	8.3	8.3
Total	3.4	31.1	26.9	11.8	18.5	8.4

^a^Likert scale, ranging from 1 (‘Looks very much like me’) to 6 (‘Does not look like me at all’)

A total of 12.6% of all men and 3.0% of all women could not identify with the portrait ‘all people being treated equally is the norm’ to rate the value 'equivalence’.

The value 'benevolence' (addressed by the portrait *‘being committed to friends and relatives is important’*) was positively rated by 100.0% of the responders in the generation aged 21–35 years and in the oldest generation. In the generation aged 36–50 years, 93.8% of the population identified with this portrait.

In the generation aged 21–35 years, about 71.3% of all responders identified with the portrait ‘looking for new things and seeking different things in life are important'. This portrait rates the value 'stimulation'. A total of 65.2% of the responders in the generation aged 36–50 years, and 58.6% of the responders in the oldest generation identified with the portrait.

The value 'tradition' is represented in the portrait *'Traditions are important’.* A total of 70.8% of men aged 51–65 years, 61.3% of men aged 36–50 years, and 57.8% of men aged 21–35 years identified with this portrait.

Work ethic was questioned using the statements of the Protestant Work Ethic Scale. A total of 43.1% of those aged 25–35 years, 20.8% of the middle group, and 13.5% of those aged 51–65-years agreed with the statement: *'I often think I would be more successful if I gave up certain pleasures'* (Table 4).

**Table 4.** Degree of identification with the work ethic portrait 'I often think I would be more successful if I gave up certain pleasures', by age group

Age group	1	2	3	4	5	6
25–35 years, %	11.7	26.3	19.0	19.7	18.2	5.1
36–50 years, %	18.8	37.5	22.9	12.5	4.2	4.2
51–65 years, %	13.5	43.2	29.7	10.8	2.7	0.0
Total, %	13.5	31.5	21.6	16.7	12.6	4.1

Likert scale ranging from 1 (‘Do not agree at all’) to 6 (‘Completely agree’)

### Qualitative focus group section

After analyses of the quantitative section above, the research group agreed on the formulation of eight statements to be submitted to focus groups. The selected statements addressed the following: the prevalence of burnout (two statements); view on work engagement (one statement); view on values and norms (four statements); and view on work ethic (one statement). Further information is available from the author on request. In the focus groups, there was an immediate consensus on certain statements. The statements on which there was no consensus in round one were fed back to the focus groups in a second round (Table 3; further information available from the author on request). A third adaptation and discussion round appeared not to be necessary.

Statement 1 addressed the age distribution of burnout: *‘With increasing age, the prevalence of burnout decreases. What do you think explains these differences?’* The two youngest age groups argued that *‘the pressure on young people is too high: they have to obtain a good diploma, find a good job, settle down and perform well on social media’* (two rounds taken to reach consensus). The older age group argued that *‘young people find it difficult to make choices, they want everything right now, and they put more pressure on themselves. Older people are better able to put problems into perspective in both their private and professional lives’* (two rounds to reach consensus).

Statement 2 addressed the sex distribution of burnout: *‘Substantially more women met the criteria for burnout compared with men. What do you think explains these differences?’* All three groups argued that still more women than men combine the care for family and housekeeping with work. The youngest generation needed two rounds to formulate a common consensus.

The third statement addressed the value ‘equivalence’: *‘Over 10% of men of all age groups do not agree with the universal equivalence principle, while a negligible number of women follow this opinion. What do you think explains this result?’* The youngest generation argued that *‘inequality between men and women is still to the advantage of men. Therefore, men do not agree with the equivalency principle because it is in their favour’* (one round to reach consensus). The middle generation argued that *‘in many families the traditional role pattern still exists where the man goes to work and the woman takes care of the household. This perpetuates inequality because men, as breadwinners, feel superior to women’* (one round to reach consensus). The oldest generation argued that ‘women have a stronger social awareness, making them more committed to equality for all’ (two rounds to reach consensus).

The fourth statement addressed commitment to friends and loved ones: *‘All responders confirmed the importance of this value, in both the youngest and the oldest generation. How do you explain these numbers?’* The younger generations argued that *‘friends and loved ones are very important to everyone over the generations, and if we can share love and suffering we feel better. That is why we tend to commit to others, to maintain these relationships’* (one round to reach consensus). The oldest generation argued that *‘the middle generation is mainly busy with their own family* [for example, children]* and thus has less time for friends. In the oldest generation, loved ones again become more important, because of needy relatives* [for example, parents]*. At the same time, the burden of their own close family decreases’* (two rounds to reach consensus).

The fifth statement addressed the work ethic: *‘With increasing age, participants felt significantly less that they had to give up pleasures in favour of work. What do you think explains such differences?’* Both younger generations argued that older people realise that they do not have to give up their pleasures to be successful (two rounds to reach consensus). The oldest generation argued that young people are confronted with many more temptations than older people and *‘there are more opportunities now than there used to be in the past. They have more fun things to giving up than older responders’* (two rounds to reach consensus).

The sixth statement addressed engagement: *‘The youngest generation seems to be far more engaged in work than the older generations. How can you explain this difference?’* The youngest generation argued that young people still want to prove themselves and that they are more interested in seeking success, while older people are more interested in seeking rest and tranquility (one round to reach consensus). The middle generation argued that older generations spend more time on private matters at the expense of work (one round to reach consensus). The oldest generation argued that young people are much more exposed to new challenges and they still have to prove themselves, which stimulates them. This leads to higher engagement in work. The older generation is more aware of which engagement is important (one round to reach consensus).

The seventh statement addressed the need of being stimulated: *‘With increasing age, the importance of striving for surprising and different things in life decreases. What do you think explains these differences?’* All generations agreed that young people still have to discover the world and gain as many new experiences as possible. The older generation has already been able to partake in all these experiences (two rounds to reach consensus).

The eighth statement addressed tradition: *'The older the male participants were, the more they adhered to tradition and family values. How can you explain these differences?’* All generations argued that it is socially acceptable for the younger generation to go their own way and that *‘you do not have to stick to the traditions of previous generations’* (one round to reach consensus).

## Discussion

### Summary

This study investigated whether the prevalence of burnout differed between generations, and whether any differences in work ethic, values, and norms presented as job demands or as resources.

The prevalence of burnout differed between the different working generations. This study showed that almost one in four of young people aged 21–35 years faced burnout. This number was remarkably higher than the 0.8%–12.9% found in the literature.^7,19–22^ One possible explanation could be that, owing to the recruitment strategy, participants were more highly educated than average. This group is more often confronted with burnout.^3,21–24^

Two job demands that young people are confronted with were higher performance pressure and difficulty making choices.^24,25^ The older generation seemed more capable putting things into perspective. This characteristic was identified as a job resource. The survey showed that almost half of the youngest generation believed they would be more successful if they gave up pleasures. This finding contrasted with the much lower number of responders aged 56–65 years who agreed with this statement. The focus groups concluded that people should not give up all the pleasant things in their personal lives to be successful at work. A good balance between work and leisure seemed to be a second job resource and serve as a protective factor against burnout. This observation was in agreement with findings in the literature.^7,20,23,26^

A third job resource was identified as tradition. The younger generation attached significantly less importance to tradition than the older generations. This finding corresponds with the literature.^16^ All generations agreed that the increased openness of society was the main explanation for this observation.

Finally, it was observed that the younger generation attached more importance to stimulation, or the search for new experiences in life. This attitude was recognised as an additional burden, acting as a job demand and thus a risk factor for burnout in the youngest generation.^9,25,27^

This research also showed that burnout was more common among women than among men. This phenomenon is confirmed in the literature.^19^ All generations identified the same stressor for this observation, namely the combination of work and family. Indeed, family stressors weigh more heavily on women than on men, particularly if a family with young children is involved.^25,28^ This observation was in line with the answers addressing equivalence; for example, women attached considerably more importance to this value than men did. The youngest generation interpreted this observation as an expression of the inequality between men and women. The middle generation recognised the stressor of the traditional role pattern. A poor work–life balance is identified as a risk factor for burnout.^25,26^ The oldest generation explained this finding by ascribing a higher social awareness to women. The authors suspect that this increased sense of equivalence acts as a job demand and is an additional risk factor for burnout.

The value of benevolence, being committed to loved ones, is highly valued by all generations and is considered as a resource protecting against burnout by all participants.^22,25^

An interesting observation was that the numbers for engagement, as the numbers for burnout, were highest in the youngest generation. Indeed, certain work requirements can also be challenging in a positive way. These challenges lead to development, growth, and (indirectly) to more work engagement.^8,9^ The youngest generation indeed argued that the younger generation wants to prove themselves more than the older generation and, therefore, becomes more involved in their work. This is in line with the higher work ethic found in the youngest generation. The middle generation suggested that older people are less committed at work because they invest more time in their private lives. The oldest generation agreed with this statement and added that the youngest generation is more stimulated by new challenges, making it easier to commit themselves.

### Strengths and limitations

This study contains several limitations. First, the web survey was distributed via the authors' social network, which explains the involvement of a higher educated working population. However, while the overall prevalence of burnout seemed higher, the age- and sex-related prevalence was in agreement with other research.^1–3^ Second, prevalence of burnout increases with level of education. Third, in this type of research, selection bias due to emotional involvement is not coincidental. Additionally, since this is a cross-sectional observation, no causal links can be made between burnout and engagement on the one hand, and job demands and resources on the other.

A strength of the study is that the theoretical links between generation and burnout, engagement and work ethic that emerged from this study do seem to correspond with those previously reported in the literature and showed a logical consistency.^1,10,22,29^ A second strength is the exploration of the quantitative data in the focus groups. With an average of one discussion round and a maximum of two rounds, participants reached consensus on the findings of the quantitative part of the study.

### Comparison with existing literature

This is the first research applying the JD-RM to different generations, and links demands and resources to intergenerational differences in work ethic, engagement, and norms and values.

### Implications for research

Further research should generalise these findings and target tailor-made interventions in the prevention of burnout. Prevention and intervention programmes should diversify their offering, addressing the particular needs of the target generation.

Burnout seems more common among younger than older people. The comments of both the younger generations on the statements addressing job demands and resources were similar. The older generation emphasises a good ability to put things into perspective, a good balance between work and leisure, and a strong sense of tradition as resources in the struggle against burnout. The younger generation has to deal with various stressors (job demands), such as increased performance pressure, the urge for stimulation, and more choice stress than the older generation. Remarkably, this generation appears to have a better work ethic and a higher commitment.
